# Myoglobin Offers Higher Accuracy Than Other Cardiac-Specific Biomarkers for the Prognosis of COVID-19

**DOI:** 10.3389/fcvm.2021.686328

**Published:** 2021-08-12

**Authors:** Jia-Sheng Yu, Ru-Dong Chen, Ling-Cheng Zeng, Hong-Kuan Yang, Hua Li

**Affiliations:** Department of Neurosurgery, Tongji Hospital, Tongji Medical College, Huazhong University of Science and Technology, Wuhan, China

**Keywords:** COVID-19, myoglobin, myocardial biomarkers, in-hospital mortality, rhabdomyolysis

## Abstract

Although sporadic studies have shown that myoglobin may have better prognostic performance than other cardiac markers in COVID-19, a comprehensive comparative study is lacking. Herein, we retrospectively analyzed the clinical and laboratory data of COVID-19 patients admitted to the Guanggu Campus of Wuhan Tongji Hospital from February 9, 2020 to March 30, 2020, intending to compare the prognostic accuracy of three commonly used cardiac markers on COVID-19 mortality. Our results revealed that abnormal increases in myocardial biomarkers were associated with a significantly increased risk of in-hospital mortality with COVID-19. Interestingly, myoglobin, a non-cardiac-specific biomarker, also expressed in skeletal myocytes, had even higher prognostic accuracy than cardiac-specific biomarkers such as high-sensitivity troponin I (hs-TnI) and creatine kinase-MB (CK-MB). More importantly, multivariate Cox analysis showed that myoglobin, rather than hs-TnI or CK-MB, was independently prognostic for in-hospital mortality in COVID-19. These results were further confirmed by subgroup analyses of patients with severe and critical illnesses and those without a history of cardiovascular disease. Our findings suggest that myoglobin may be a reliable marker of illness reflecting general physiological disturbance and help to assess prognosis and treatment response in patients with COVID-19.

## Introduction

The coronavirus disease 2019 (COVID-19) pandemic caused by severe acute respiratory syndrome coronavirus 2 (SARS-CoV-2) has become a global health concern (1). Despite the advent of vaccines that have slowed the spread of the virus, COVID-19 may likely become an endemic disease that will coexist with humans for a long time. Patients with COVID-19 may deteriorate to multi-organ dysfunction including myocardial failure (2–5). Increases in troponin and creatine kinase-MB (CK-MB), two of the most sensitive indicators of myocardial injury, have been demonstrated to be associated with a more severe clinical course and to correlate with poorer outcomes in COVID-19 (6–9). However, in our previous study and studies by other investigators, multifactorial analysis combining most clinical indicators showed that hypersensitive troponin I (hs-TnI) and CK-MB were not independent predictors of mortality in COVID-19. In contrast, myoglobin (MYO), a non-cardiac-specific indicator also expressed in skeletal muscle cells, was independent prognostic (10–15). The reasons underlying this curious phenomenon were not fully understood, but a comprehensive comparison of the predictive capabilities of myocardial biomarkers can help clarify the mechanisms involved and may contribute to our understanding of the significance of cardiac injury in COVID-19. This study retrospectively analyzed the clinical and laboratory data of patients with COVID-19 admitted to the Guanggu Campus of Wuhan Tongji Hospital between February 9, 2020 and March 30, 2020, to compare the prognostic accuracy of three commonly used cardiac markers for in-hospital mortality of COVID-19. Exploring their prognostic roles is of great significance for identifying useful biomarkers for early diagnosis, prognosis, and therapeutic response assessment in clinical practice.

## Methods

### Study Design and Participants

This study was a retrospective study conducted in the Guanggu Campus of Tongji hospital (Wuhan), Tongji Medical College, Huazhong University of Science and Technology, which was a designated hospital for patients with COVID-19 during the outbreak of novel coronavirus disease in Wuhan, China. The diagnosis of COVID-19 was confirmed by RNA testing of the SARS-CoV-2 in the on-site clinical laboratory according to the WHO interim guidance. 1,284 patients with laboratory-confirmed COVID-19 between February 9, 2020 and March 30, 2020 were initially enrolled. Patients aged <18 years or lacking cardiac biomarker results (e.g., MYO, CK-MB, and hs-TnI) were excluded. Ultimately, 1,229 patients were enrolled in this study. The study was approved by the ethics committee of Tongji hospital, Huazhong University of Science and Technology (TJ-IRB20210317). Written informed consent was waived due to the use of de-identified retrospective data.

### Data Collection and Follow-Up

All clinical, laboratory and outcome data were available and were collected according to the electronic medical records using a standardized data collection form. Data collection for early laboratory results was defined using the first examination at admission (within 24 h of admission). Data collection for the late-stage laboratory results was defined using the last assessment during hospitalization. All data were verified by two physicians blinded to the patient's identity.

Demographic characteristics, medical history, and physical examination findings at admission, and early and late laboratory results during hospitalization were collected. Demographic characteristics obtained for the study included age and gender. Medical history included hypertension (HP), diabetes (DM), chronic liver disease (CLD), coronary heart disease (CHD), cerebrovascular disease, chronic kidney disease (CKD), chronic obstructive pulmonary disease (COPD), and cancer. Physical examination findings included first body temperature, respiratory rate, pulse rate, systolic blood pressure (SBP), diastolic blood pressure (DBP), and saturation of pulse oxygen (SpO_2_). Laboratory findings included hs-TnI, CK-MB, MYO, neutrophil (NEU), lymphocyte (LYM), high sensitivity C-reactive protein (hs-CRP), interleukin 6 (IL-6), D-dimer, fibrinogen (FIB), alanine aminotransferase (ALT), albumin (ALB), creatinine (Cr), estimated glomerular filtration rate (EGFR), and glucose (GLU) levels. All clinical samples were tested in the same laboratory using the same standard.

All patients were followed up from admission to discharge to observe the risks of in-hospital mortality. Survival time was defined as the time from hospital admission to discharge or death. The follow-up data were obtained from reviewing medical records by trained researchers using a double-blind method.

### Myocardial Injury Biomarkers

In this study, we focused on three commonly used biomarkers of myocardial injury (hs-TnI, CK-MB, and MYO). At our institution, these three biomarkers have been integrated as a test package into the laboratory test panel for COVID-19 patients. Serum levels of hs-TnI, CK-MB, and MYO were measured by chemiluminescence microparticle immunoassays on the Architect i2000SR platform (Abbott Laboratories, Chicago, IL) according to manufactures' instructions. The values were considered elevated if they were above the upper limit of normal (ULN) levels, which were defined as the 99th upper percentile of the biomarker distribution in the normal population. According to our laboratory normal ranges, the ULN levels of hs-TnI, CK-MB, and MYO were 34.2 pg/ml, 5.2 ng/ml, and 154.9 ng/ml, respectively, in male patients, and were 15.6 pg/ml, 3.1 ng/ml, and 106 ng/ml in female patients.

### Clinical Classifications

According to the Guidance for Corona Virus Disease 2019: Prevention, Control, Diagnosis, and Management edited by the National Health Commission of the People's Republic of China (16), all cases were identified into four categories of mild cases, ordinary cases, severe cases, and critical cases based on the clinical conditions at the time of admission. (1) Mild cases: the clinical symptoms are mild, and no pneumonia manifestation can be found in imaging. (2) Ordinary cases: patients have symptoms like fever and respiratory tract symptoms, and pneumonia manifestation can be seen in imaging. (3) Severe cases: meeting any of the following: respiratory distress, RR ≥ 30 breaths/min; the oxygen saturation is <93% at a rest state; arterial partial pressure of oxygen (PaO_2_)/oxygen concentration (FiO_2_) ≤ 300 mmHg (1 mmHg = 0.133 kPa). Patients with >50% lesions progression within 24 to 48 h in pulmonary imaging were treated as severe cases. (4) Critical cases: meeting any of the following: respiratory failure occurs, mechanical ventilation is needed; shock occurs; or complicated with other organ failures that require monitoring and treatment in the intensive care unit.

### Statistical Analysis

Continuous variables were presented as mean ± standard deviation or median (inter-quartile range, IQR), as appropriate. Categorical variables were presented as *n* (%). Event frequencies were compared by chi-square test. Differences in cardiac biomarker levels between the early- and late-stage groups were compared using the Wilcoxon signed-ranks test (two-tailed). Other comparisons between the two groups were made using the independent samples *t*-test (normally distributed continuous variables) or the Mann-Whitney *U*-test (non-normally distributed continuous variables).

To better understand the overall performance of myocardial biomarkers, we performed both standard receiver-operating characteristic (ROC) analysis and time-dependent ROC curve analysis (17, 18). The area under the ROC curve (AUC) was calculated to evaluate the performance of each biomarker. The optimal cut-off point was assessed by standard ROC analysis and determined using the maximization of the Youden's index. Time-dependent AUC curves with 95% confidence intervals (CIs) were performed to compare the prognostic accuracy of cardiac biomarkers.

Cumulative survival curves for in-hospital death were assessed using Kaplan-Meier product-limit estimation with log-rank tests. Univariate Cox proportional hazards regression and mixed-effects Cox models were used to calculate hazard ratios (HRs) for risk factors.

Multivariate Cox proportional hazards model analysis was performed to identify independent prognostic factors associated with in-hospital mortality in patients with COVID-19. Considering the possible collinearity issues, the least absolute shrinkage and selection operator (LASSO) regression was previously performed to screen potential prognostic factors (19). Variables with non-zero coefficients in the LASSO regression were selected for further proportional hazard assumption (Schoenfeld test) to assess the applicability of the variables to the multivariate Cox model (20). If the proportional hazard assumption was not violated (*p* > 0.05), the multivariate Cox proportional hazard model was then performed. Only variables with a *p*-value < 0.05 in the multivariate analysis were considered as independent prognostic factors. The predictive performance of the multivariate Cox model was evaluated by Harrell's concordance index (*C*-index).

The cases with missing biomarker data were excluded listwise with statistics software. Statistical analyses were performed using SPSS 22.0 (SPSS, Chicago, IL, USA) and R software version 3.6.3 (http://www.r-project.org). *p*-value < 0.05 was considered statistically significant.

## Results

### Baseline Characteristics and Laboratory Results of Patients Stratified by Mortality

Of the 1,229 eligible patients, 66 (5.4%) died during follow-up. Table 1 presents the basic clinical characteristics and physical examination findings for the overall study population and for patients stratified by mortality. Patients who died were older, more likely to be male, and more likely to have a history of stroke and chronic kidney disease than those who were alive. Besides, they had lower SpO_2_ and DBP levels and higher body temperature and respire rate levels at admission (Table 1).

**Table 1:** Baseline characteristics of the overall study population and patients stratified based on mortality.

**Characteristics**	**Total (*n* = 1229)**	**Alive (*n* = 1163)**	**Died (*n* = 66)**	***P*-value**
Age (yrs.), median (IQR)	62 (51-70)	61 (50-69)	71 (67-81)	** <0.001**
Male, *n* (%)	588 (47.8)	541 (46.5)	47 (71.2)	** <0.001**
**Comorbidities**, ***n*****(%)**				
History of HP-*n* (%)	433 (35.2)	404 (34.7)	29 (43.9)	0.128
History of DM-*n* (%)	207 (16.8)	193 (16.6)	14 (21.2)	0.330
Chronic liver disease-*n* (%)	20 (1.6)	19 (1.6)	1 (1.5)	0.941
History of CHD-*n* (%)	95 (7.7)	88 (7.6)	7 (10.6)	0.368
Stroke history-*n* (%)	49 (4.0)	42 (3.6)	7 (10.6)	**0.005**
Chronic kidney disease-*n* (%)	31 (2.5)	25 (2.1)	6 (9.1)	** <0.001**
History of COPD-*n* (%)	10 (0.8)	9 (0.8)	1 (1.5)	0.514
Cancer-*n* (%)	41 (3.3)	37 (3.2)	4 (6.1)	0.205
**Physical examination on admission, median (IQR)**				
Temperature (°C)	36.5 (36.2-36.9)	36.5 (36.2-36.9)	36.6 (36.4-37.1)	**0.021**
Pulse (/min)	89 (80-100)	89 (80-100)	92 (83-102)	0.275
Respire (/min)	20 (19-22)	20 (19-22)	21 (20-30)	**0.001**
SBP (mmHg)	132 (120-145)	132 (120-145)	130 (117-144)	0.336
DBP (mmHg)	80 (72-90)	80 (73-90)	78 (70-87)	0.106
SpO_2_ (%)	97 (95-98)	97 (95-98)	92 (88-97)	** <0.001**
Hospital stay-days, median (IQR)	21 (14-32)	21 (14-33)	14 (9-21)	** <0.001**

*p-values were calculated between the alive and died groups by Mann-Whitney U-test or chi-square test, as appropriate. IQR, interquartile range; HP, hypertension; DM, diabetes; CHD, coronary heart disease; COPD, chronic obstructive pulmonary disease; SBP, systolic blood pressure; DBP, diastolic blood pressure; SpO_2_, percutaneous oxygen saturation.*

*The bold p values mean that they are of significance in statistics*.

Table 2 shows the levels of early and late laboratory parameters in the overall study population, survivors, and non-survivors. Non-survivors had lower LYM, ALB, and EGFR and higher levels of hs-TnI, CK-MB, MYO, NEU, hs-CRP, IL-6, D-dimer, ALT, Cr, and GLU in the early stages of hospitalization compared to survivors. These trends could also be found in the later levels of laboratory results (Table 2).

**Table 2:** Laboratory findings at the early and late stages of hospitalization in overall study patients and patients stratified based on mortality.

**Characteristics**	**N**	**Total**	**Alive**	**Died**	***P*-value**
**Laboratory results at early stage, median (IQR)**					
Hs-TnI (pg/ml)	1,229	3.1 (1.9-8.0)	2.8 (1.9-7.4)	25.8 (8.0-223.6)	** <0.001**
CK-MB (ng/ml)	1,229	0.7 (0.5-1.2)	0.7 (0.4-1.1)	2.4 (1.0-4.4)	** <0.001**
MYO (ng/ml)	1,229	35.6 (26.1-59.6)	34.5 (25.6-53.9)	169.2 (95.7-368.5)	** <0.001**
NEU (10∧9/L)	1,229	3.68 (2.67-5.17)	3.59 (2.63-4.97)	7.50 (4.91-11.87)	** <0.001**
LYM (10∧9/L)	1,229	1.34 (0.94-1.79)	1.38 (1.00-1.82)	0.60 (0.43-0.88)	** <0.001**
Hs-CRP (mg/L)	1,228	5.2 (1.2-37.1)	4.1 (1.1-31.1)	89.1 (44.9-144.5)	** <0.001**
IL6 (pg/ml)	1,095	3.76 (1.76-11.92)	3.50 (1.66-9.66)	58.95 (24.55-167.55)	** <0.001**
D-dimer (μg/ml FEU)	1,214	0.56 (0.24-1.36)	0.51 (0.23-1.14)	5.61 (1.71-21.00)	** <0.001**
FIB (g/L)	1,221	4.06 (3.20-5.41)	4.02 (3.20-5.35)	4.79 (2.94-6.09)	0.081
ALT (U/L)	1,229	21.0 (13.0-36.0)	20.0 (13.0-35.0)	26.0 (17.8-44.5)	**0.004**
ALB (g/L)	1,229	37.4 (33.3-41.5)	37.6 (33.8-41.7)	31.3 (27.8-34.6)	** <0.001**
Cr (μmol/L)	1,229	67 (56-82)	67 (56-80)	88 (66-117)	** <0.001**
EGFR (ml/min/1.73m∧2)	1,229	93.2 (79.3-103.5)	93.7 (80.6-104.0)	67.4 (49.1-89.4)	** <0.001**
GLU (mmol/L)	1,223	5.57 (4.97-7.02)	5.51 (4.94-6.83)	7.24 (5.96-10.30)	** <0.001**
**Laboratory results at late stage, median (IQR)**					
Hs-TnI (pg/ml)	1,229	2.3 (1.9-5.8)	2.2 (1.9-5.0)	144.9 (31.3-544.9)	** <0.001**
CK-MB (ng/ml)	1,229	0.6 (0.4-1.0)	0.6 (0.4-0.9)	4.8 (1.9-10.2)	** <0.001**
MYO (ng/ml)	1,229	30.2 (23.5-43.5)	29.5 (23.1-39.9)	672.7 (279.9-1200.0)	** <0.001**
NEU (10∧9/L)	1,229	3.25 (2.51-4.19)	3.16 (2.49-4.03)	11.09 (6.8-17.61)	** <0.001**
LYM (10∧9/L)	1,229	1.58 (1.25-1.96)	1.61 (1.29-1.99)	0.44 (0.29-0.77)	** <0.001**
Hs-CRP (mg/L)	1,228	1.6 (0.7-5.3)	1.5 (0.6-4.2)	114.7 (72.4-198.0)	** <0.001**
IL6 (pg/ml)	1,094	3.14 (1.57-7.75)	2.96 (1.51-6.35)	377.60 (75.19-1698.50)	** <0.001**
D-dimer (μg/ml FEU)	1,215	0.40 (0.22-0.94)	0.37 (0.22-0.79)	6.68 (3.34-16.05)	** <0.001**
FIB (g/L)	1,221	3.59 (2.99-4.43)	3.57 (2.99-4.30)	4.52 (3.02-5.84)	**0.003**
ALT (U/L)	1,226	19.0 (12.0-30.0)	19.0 (12.0-30.0)	29.0 (18.0-48.0)	** <0.001**
ALB (g/L)	1,216	39.6 (36.5-42.0)	39.8 (36.8-42.1)	31.1 (28.1-34.3)	** <0.001**
Cr (μmol/L)	1,226	68 (57-81)	67 (57-80)	121 (76-166)	** <0.001**
EGFR (ml/min/1.73m∧2)	1,223	93.2 (80.6-103.0)	93.8 (82.4-103.4)	47.2 (32.2-82.6)	** <0.001**
GLU (mmol/L)	1,223	5.25 (4.79-6.29)	5.21 (4.78-6.06)	8.51 (6.32-11.32)	** <0.001**

*p-values were calculated between alive and died groups by Mann-Whitney U-test. n, number; IQR, interquartile range; Hs-TnI, high sensitivity troponin-I; CK-MB, creatine kinase-MB; MYO, myoglobin; NEU, neutrophil; LYM, lymphocytes; Hs-CRP, high sensitivity C-reactive protein; IL6, interleukin 6; FIB, fibrinogen; ALT, alanine aminotransferase; ALB, albumin; Cr, creatinine; EGFR, estimated glomerular filtration rate; GLU, glucose.*

*The bold p values mean that they are of significance in statistics*.

In terms of dynamic characteristics, the levels of hs-TnI, CK-MB, MYO, NEU, hs-CRP, IL-6, D-dimer, FIB, ALT, and GLU in survivors decreased significantly in the late stage of the disease. In contrast, in non-survivors, their levels increased significantly in the late stage (see Supplementary Table 1).

### Myocardial Injury Biomarker Levels in COVID-19 Patients

**Figure 1:**
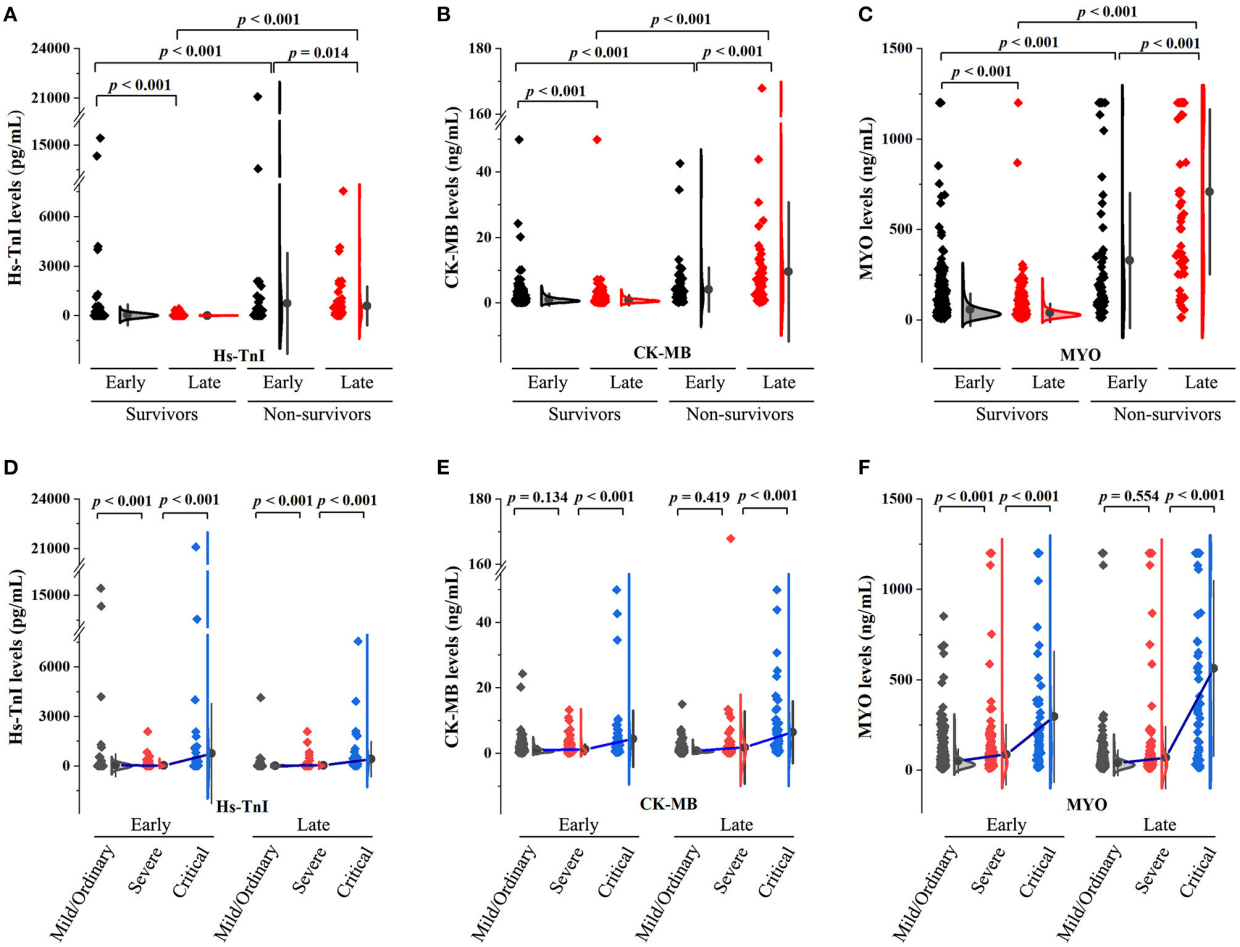
Summary of myocardial biomarker levels in patients with COVID-19. **(A–C)** The early- and late-stage levels of myocardial biomarkers in patients stratified by mortality. **(D–F)** The early- and late-stage levels of myocardial biomarkers in patients stratified by clinical classifications.

Figures 1A-C demonstrate the levels of myocardial injury biomarkers. As shown, myocardial biomarker levels were significantly higher in non-survivors compared to survivors in both early and late stages. In terms of dynamic changes, myocardial biomarker levels were significantly decreased in survivors while elevated in non-survivors.

There were 18 (1.5%), 912 (74.2%), 231 (18.8%), and 68 (5.5%) patients with mild, ordinary, severe, and critical conditions, respectively. Myocardial biomarker levels in patients stratified according to clinical classification are shown in Figures 1D-F. Patients in the severe group had higher levels of hs-TnI and MYO in the early stages and higher levels of hs-TnI in the late stages compared to the mild/ordinary group. Patients in the critical group had significantly higher myocardial biomarker levels in both early and late stages than in the severe group.

The percentages of patients with myocardial marker levels below the cut-off (for definition, see later section), between the cut-off and ULN, and above the ULN are illustrated in Supplementary Figures 1A,B. The percentages of deaths in patients with myocardial marker levels below the cut-off, between the cut-off and ULN, and above the ULN were shown in Supplementary Figures 1C,D.

Supplementary Figure 2 shows the correlation matrix heat map for 16 clinical parameters in the early and late stages of the disease. MYO levels were significantly correlated with creatinine, EGFR, hs-CRP, IL-6, hs-TnI, CK-MB, and age. Hs-TnI levels were significantly correlated with ALB, LYM, EGFR, hs-CRP, IL-6, and D-dimer.

### Associations of Elevated Myocardial Biomarker Levels With In-hospital Mortality of COVID-19

To evaluate the associations between in-hospital mortality of COVID-19 and covariates including myocardial injury markers, we performed Cox regression analysis where the Wald **χ^2^** and *p*-values were calculated. After adjusting for age, gender, and comorbidities (HP, DM, CLD, CHD, CKD, COPD, stroke, and cancer), hs-TnI, CK-MB, and MYO were significantly associated with in-hospital mortality of COVID-19. Notably, the Wald **χ^2^** values for MYO were relatively higher than those for hs-TnI and CK-MB (Supplementary Table 2).

To assess the association of each myocardial marker elevation above ULN with in-hospital mortality, Cox regression-derived hazard ratios were calculated. After adjusting for age, gender, and comorbidities, the HRs for the risks of in-hospital mortality for elevated early-stage levels of Hs-cTnI, CK-MB, and MYO were 7.47 (95% CI, 4.39-12.72, *p* < 0.001), 10.37 (95% CI, 5.87-18.30, *p* < 0.001), and 7.96 (95% CI, 4.75-13.35, *p* < 0.001). Adjusted HRs for elevated late-stage levels of Hs-cTnI, CK-MB, and MYO were 36.35 (95% CI, 20.13-65.66, *p* < 0.001), 27.37 (95% CI, 16.19-46.28, *p* < 0.001), and 77.54 (95% CI, 39.09-153.82, *p* < 0.001) (see Supplementary Table 3).

Kaplan-Meier curves between groups categorized by ULN are shown in Supplementary Figure 3. As illustrated, patients with hs-TnI, CK-MB, and MYO levels above the ULN had significantly decreased survival rates.

### Prognostic Performance of Myocardial Injury Biomarkers in Predicting In-hospital Mortality of COVID-19

The standard ROC curves were conducted to compare the relative accuracy, sensitivity, specificity, and positive and negative predictive value of each biomarker. The best cut-off values were calculated based on the ROC analysis. Our results show that using the cut-off values determined by the ROC curves improved prognostic accuracy over the use of ULN values.

Table 3 summarizes the overall performance of the myocardial biomarkers. When incorporating the early levels of myocardial biomarkers, the AUC was 0.89 (95% CI 0.84-0.94) for MYO, 0.84 (95% CI 0.78-0.89) for hs-TnI, and 0.78 (95% CI 0.72-0.85) for CK-MB. The optimal cut-offs were 80.8 ng/ml for MYO with a sensitivity of 84.8% and a specificity of 86.2%, 7.9 pg/ml for hs-TnI with a sensitivity of 77.3% and a specificity of 77.4%, and 1.2 ng/ml for CK-MB with a sensitivity of 71.2% and a specificity of 76.2% (Table 3).

**Table 3:** Overall performance of the myocardial biomarkers according to the standard ROC analysis.

	**Early stage**				**Late stage**		
	**Hs-TnI**	**CK-MB**	**MYO**		**Hs-TnI**	**CK-MB**	**MYO**
N	1,229	1,229	1,229		1,229	1,229	1,229
Mortality	66	66	66		66	66	66
AUC (95% CI)	0.84 (0.78-0.89)	0.78 (0.72-0.85)	0.89 (0.84-0.94)		0.94 (0.90-0.98)	0.92 (0.87-0.96)	0.96 (0.92-1.00)
cut-off	7.9 pg/ml	1.2 ng/ml	80.8 ng/ml		15.7 pg/ml	1.5 ng/ml	98.0 ng/ml
Sensitivity (95% CI)	0.77 (0.65-0.87)	0.71 (0.59-0.82)	0.85 (0.74-0.93)		0.89 (0.79-0.96)	0.85 (0.74-0.93)	0.91 (0.81-0.97)
Specificity (95% CI)	0.77 (0.75-0.80)	0.76 (0.74-0.79)	0.86 (0.84-0.88)		0.94 (0.93-0.96)	0.91 (0.89-0.93)	0.96 (0.95-0.97)
PPV (95% CI)	0.16 (0.14-0.27)	0.15 (0.13-0.23)	0.26 (0.23-0.43)		0.48 (0.41-0.70)	0.35 (0.31-0.54)	0.56 (0.49-0.78)
NPV (95% CI)	0.98 (0.97-0.99)	0.98 (0.96-0.98)	0.99 (0.98-0.99)		0.99 (0.99-1.00)	0.99 (0.98-0.99)	1.00 (0.99-1.00)
DLR.Positive (95% CI)	3.42 (2.89-4.05)	2.99 (2.49-3.60)	6.13 (5.14-7.31)		16.00 (12.45-20.55)	9.58 (7.76-11.83)	22.50 (16.83-30.07)
DLR.Negative (95% CI)	0.29 (0.19 - 0.46)	0.38 (0.26 - 0.55)	0.18 (0.10-0.31)		0.11 (0.06-0.23)	0.17 (0.09-0.29)	0.10 (0.04-0.20)
FP	263	277	161		65	103	47
FN	15	19	10		7	10	6

*N, number; AUC, area under the ROC curves; CI, confidence interval; PPV, positive predictive value; NPV, negative predictive value; DLR, diagnostic likelihood ratio; FP, false positive; FN, false negative; Hs-TnI, high sensitivity troponin-I; CK-MB, creatine kinase-MB; MYO, myoglobin*.

When incorporating the late levels of myocardial biomarkers for ROC analysis, the AUCs were 0.96 (95% CI 0.92-1.00) for MYO, 0.94 (95% CI 0.90-0.98) for hs-TnI, and 0.92 (95% CI 0.87-0.96) for CK-MB, respectively. The optimal cut-off point was 98.0 ng/ml for MYO with a sensitivity of 90.9% and a specificity of 96.0%, 15.7 pg/ml for hs-TnI with a sensitivity of 89.4% and a specificity of 94.4%, and 1.5 ng/ml for CK-MB with a sensitivity of 84.8% and a specificity of 91.1% (Table 3).

**Figure 2:**
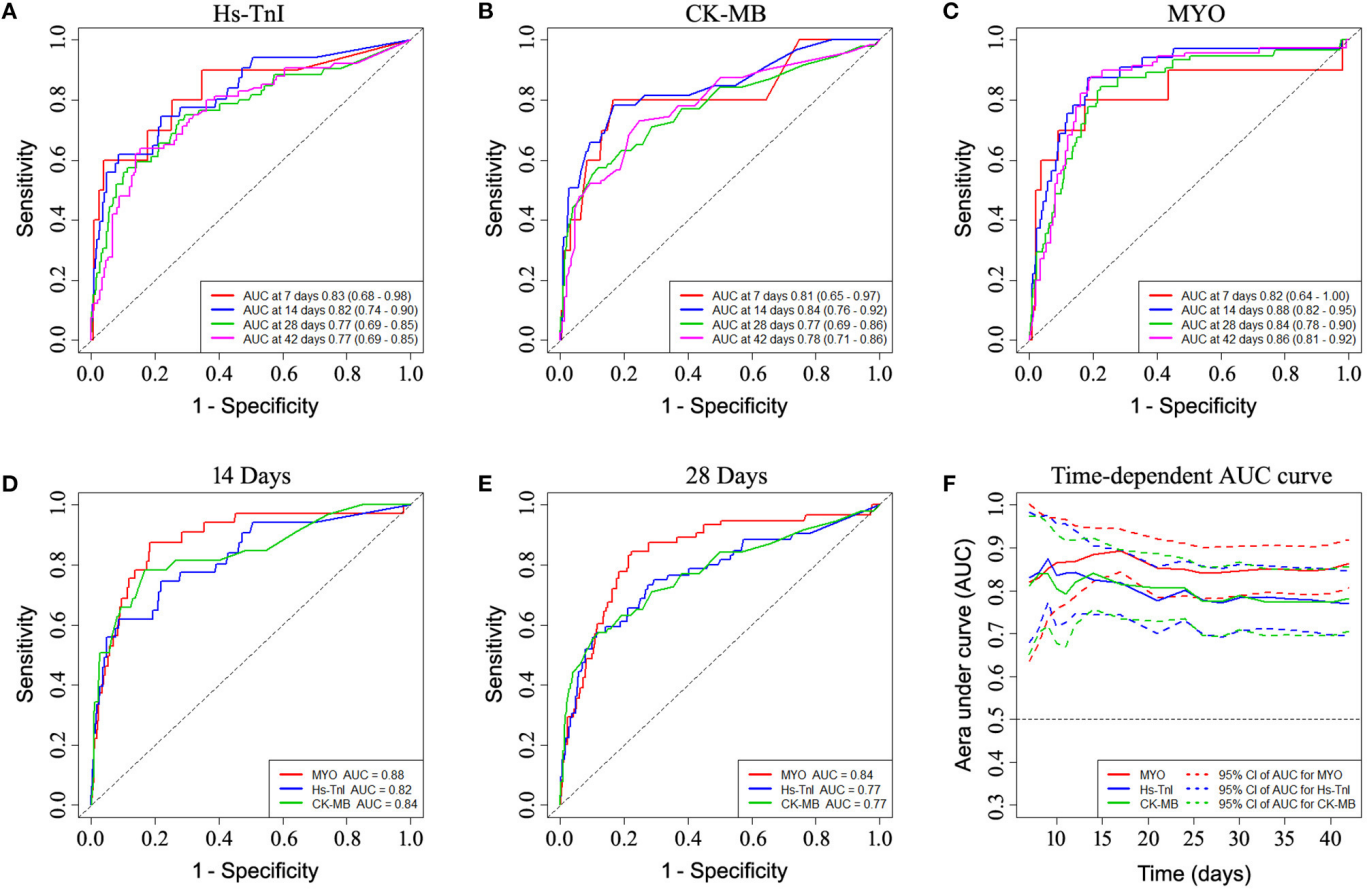
Time-dependent ROC curve analysis based on the early-stage levels of myocardial biomarkers showing the prognostic performance of myocardial biomarkers. **(A–C)** ROC performance of myocardial biomarkers at different times during follow-up. **(D,E)** Comparison of the prognostic performance of each biomarker in predicting 14-day and 28-day mortality. **(F)** Time-dependent AUC curves of myocardial markers showed superior AUC values for myoglobin over hs-TnI and CK-MB throughout the follow-up period. ROC, receiver operating characteristic curve; AUC, area under curve.

Time-dependent ROC curve analysis was performed based on early levels of myocardial biomarkers to better understand the performance of each myocardial biomarker (see Figure 2). The AUC values for predicting 14-day survival for MYO, hs-TnI, and CK-MB were 0.88, 0.82, and 0.84, respectively (Figure 2D). For predicting 28-day survival, the AUC values for MYO, hs-TnI, and CK-MB were 0.84, 0.77, and 0.77, respectively (Figure 2E). The time-dependent AUC curves for myocardial markers are shown in Figure 2F. As shown, the AUC values of MYO were superior to those of hs-TnI and CK-MB throughout the follow-up period (Figure 2F).

Patients with biomarker levels above the newly established cut-off value had a significantly higher risk of in-hospital mortality than patients with biomarker levels below the cut-off value. After adjusting for confounders including age, sex, comorbidities (HP, DM, CLD, CHD, CKD, COPD, stroke, and cancer), and disease severity indicators (hs-CRP, D-dimer, NEU) in a multivariate Cox model, the HRs for risk of mortality with increased early levels of hs-TnI, CK-MB, and MYO were 2.31 (95% CI, 1.20-4.48, *p* = 0.013), 1.82 (95% CI, 1.01-3.31, *p* = 0.048), and 8.31 (95% CI, 3.94-17.52, *p* < 0.001), respectively. Adjusted HRs for late levels of hs-TnI, CK-MB, and MYO were 18.19 (95% CI, 7.87-42.05, *p* < 0.001), 8.22 (95% CI, 3.81-17.71, *p* < 0.001), and 34.41 (95% CI, 14.15-83.68, *p* < 0.001), respectively (Table 4).

**Table 4:** Associations of increased myocardial marker levels above cut-offs with in-hospital mortality of COVID-19.

		**Crude**			**Model 1**			**Model 2**	
		**HR (95% CI)**	***P*-value**		**HR (95% CI)**	***P*-value**		**HR (95% CI)**	***P*-value**
Early-stage levels	**Hs-TnI**
	≤ cut-off	Ref			Ref			Ref	
	>cut-off	7.85 (4.46-13.81)	<0.001		5.34 (2.89-9.88)	<0.001		2.31 (1.20-4.48)	0.013
	**CK-MB**
	≤ cut-off	Ref			Ref			Ref	
	>cut-off	5.98 (3.60-9.92)	<0.001		3.73 (2.18-6.37)	<0.001		1.82 (1.01-3.31)	0.048
	**MYO**
	≤ cut-off	Ref			Ref			Ref	
	>cut-off	23.88 (12.18-46.82)	<0.001		19.36 (9.65-38.84)	<0.001		8.31 (3.94-17.52)	<0.001
Late-stage levels	**Hs-TnI**
	≤ cut-off	Ref			Ref			Ref	
	>cut-off	67.15 (32.05-140.68)	<0.001		63.54 (28.93-139.57)	<0.001		18.19 (7.87-42.05)	<0.001
	**CK-MB**
	≤ cut-off	Ref			Ref			Ref	
	>cut-off	40.99 (21.91-76.69)	<0.001		31.46 (16.53-59.87)	<0.001		8.22 (3.81-17.71)	<0.001
	**MYO**
	≤ cut-off	Ref			Ref			Ref	
	>cut-off	116.72 (53.14-256.36)	<0.001		108.64 (47.23-249.88)	<0.001		34.41 (14.15-83.68)	<0.001

*The variables were categorized into two groups according to the cut-off of each biomarker. The biomarkers were included as dichotomous variables in the univariable COX regression analysis.*

*Model 1: Adjusted for age, sex, and co-existing diseases (hypertension, diabetes, coronary heart disease, chronic obstructive pulmonary disease, chronic liver disease, stroke history, chronic kidney disease, and cancer history).*

*Model 2: Model 1 plus variables related to disease severity (neutrophil count, D-Dimer, and high sensitivity C-reactive protein).*

*HR, hazard ratio; CI, confidence interval; Hs-TnI, high sensitivity troponin-I; CK-MB, creatine kinase-MB; MYO, myoglobin*.

**Figure 3:**
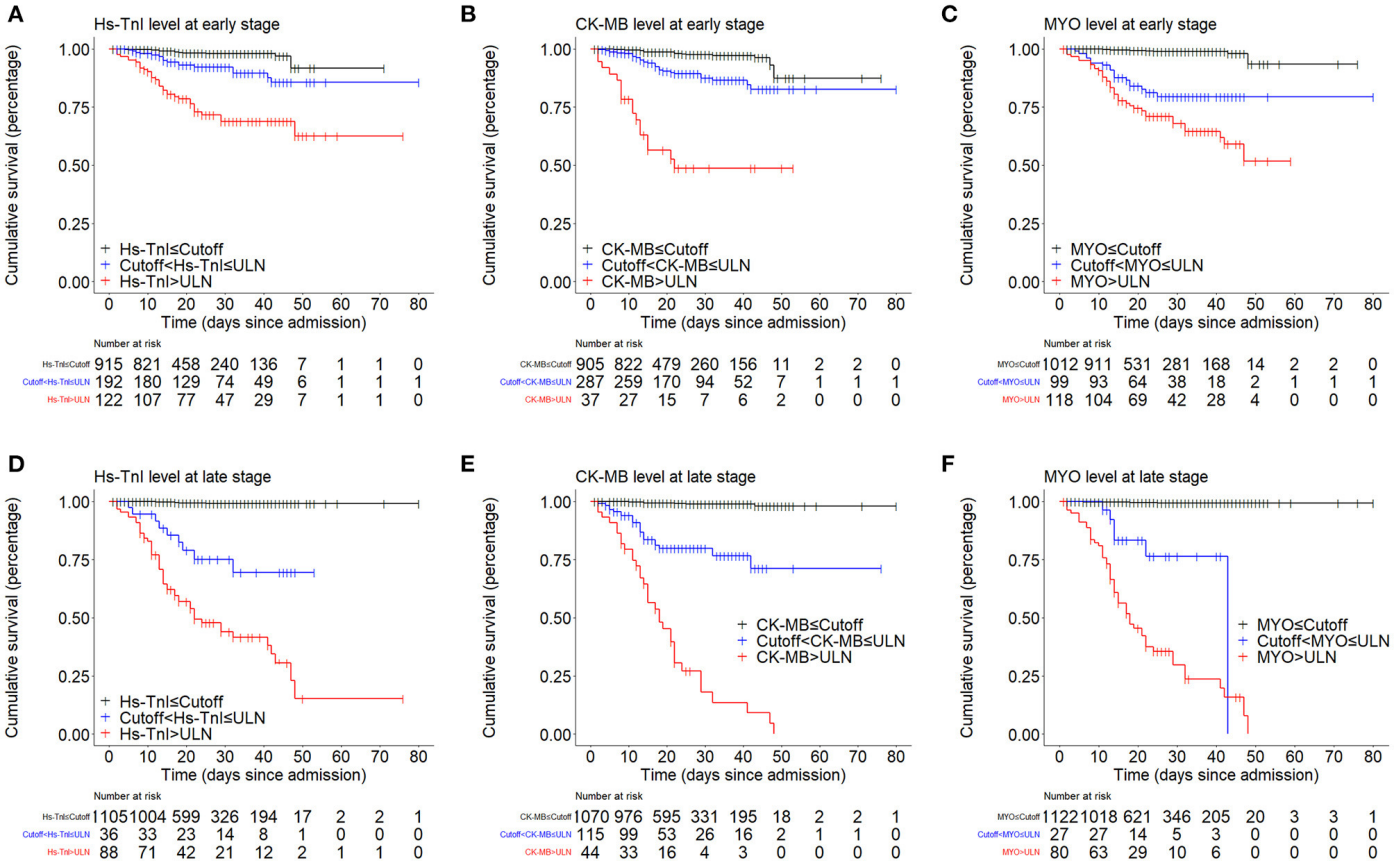
Kaplan-Meier curves showing the cumulative survival rates of patient groups divided by cut-off and ULN. **(A–C)** Kaplan-Meier curve analysis based on the early-stage levels of myocardial biomarkers. **(D–F)** Kaplan-Meier curve analysis based on the late-stage levels of myocardial biomarkers. ULN, upper limit of normal.

Kaplan-Meier curves showed an early separation of mortality curves for patients with biomarker levels above the ULN and patients with biomarker levels between the cut-off and the ULN, indicating a significantly increased risk of death in these patients (Figure 3).

Overall, these results suggest that all three commonly used cardiac biomarkers are significantly associated with in-hospital mortality. MYO provided better prognostic performance than hs-TnI and CK-MB in predicting mortality of COVID-19.

### MYO, Rather Than hs-TnI or CK-MB, Was an Independent Prognostic Factor Associated With In-hospital Mortality of COVID-19

To identify independent prognostic factors associated with COVID-19 mortality, we further developed multivariate Cox models. Potential confounders including age, sex, comorbidities (HP, DM, CLD, CHD, CKD, COPD, stroke, and cancer), physical examination on admission (temperature, respiratory rate, pulse, SBP, DBP, and SpO_2_), and laboratory parameters (hs-TnI, CK-MB, MYO, NEU, LYM, hs-CRP, IL-6, D-dimer, FIB, ALT, ALB, Cr, EGFR, and GLU) were controlled for. LASSO regression was previously performed to address possible collinearity issues.

**Figure 4:**
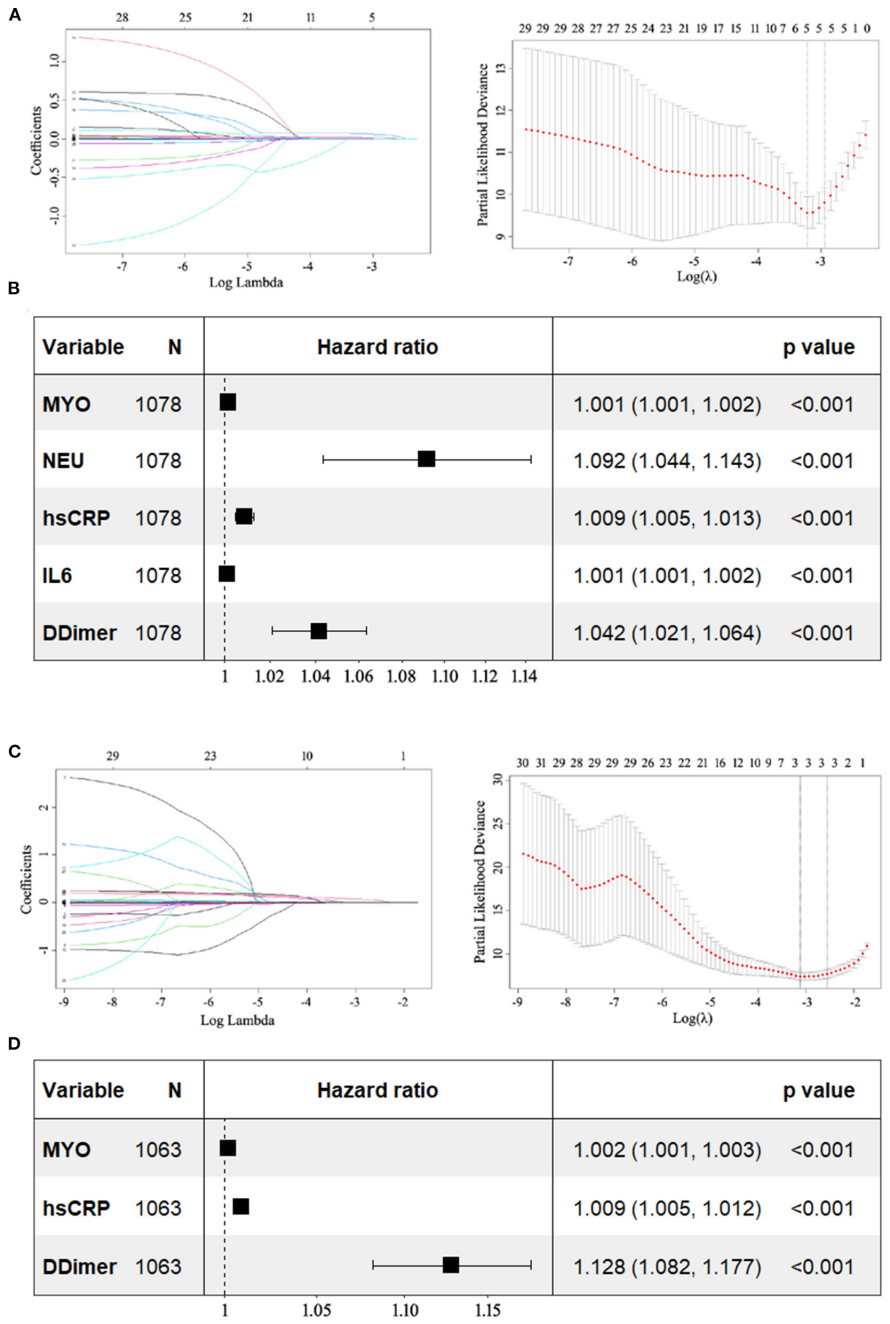
Forest plots showing the results of multivariate Cox analysis. The potential prognostic factors included in the multivariate analysis were previously identified by LASSO regression. **(A)** LASSO coefficient profiles of the 30 variables including age, sex, comorbidities, physical examinations, and early-stage results of laboratory parameters. Dotted vertical lines were drawn at the optimal values by using the minimum criteria and the 1 SE of the minimum criteria (the 1-SE criteria). Five variables with non-zero coefficients (MYO, NEU, hs-CRP, IL-6, and D-dimer) were identified. **(B)** In multivariate analysis, all five variables had *p*-values < 0.05. **(C)** LASSO coefficient profiles of the 30 variables including age, sex, comorbidities, physical examinations, and late-stage results of laboratory parameters. Three variables (MYO, hs-CRP, and D-dimer) with non-zero coefficients were identified. **(D)** In multivariate analysis, they all had *p*-values less than 0.05. LASSO, least absolute shrinkage and selection operator; MYO, myoglobin; NEU, neutrophil; hs-CRP, high sensitivity C-reactive protein; IL-6, interleukin 6.

When incorporating variables including age, sex, comorbidities, physical examinations, and early-stage laboratory results in the LASSO regression, five variables (MYO, NEU, hs-CRP, IL-6, and D-dimer) with non-zero coefficients were identified (see Figure 4A). They all had *p*-values < 0.05 in the multivariate model, suggesting an independent prognostic effect of early levels of MYO, NEU, hs-CRP, IL-6, and D-dimer on COVID-19 mortality (see Figure 4B).

Three variables (MYO, hs-CRP, and D-dimer) were identified when incorporating variables including age, sex, comorbidities, physical examinations, and late-stage laboratory results in the LASSO regression (Figure 4C). They were all found to have *p*-values < 0.05, indicating that late levels of MYO, hs-CRP, and D-dimer had an independent prognostic effect on mortality in COVID-19 (see Figure 4D).

### Subgroup Analyses Confirming the Strong Prognostic Ability of MYO for In-hospital Mortality in COVID-19

**Figure 5:**
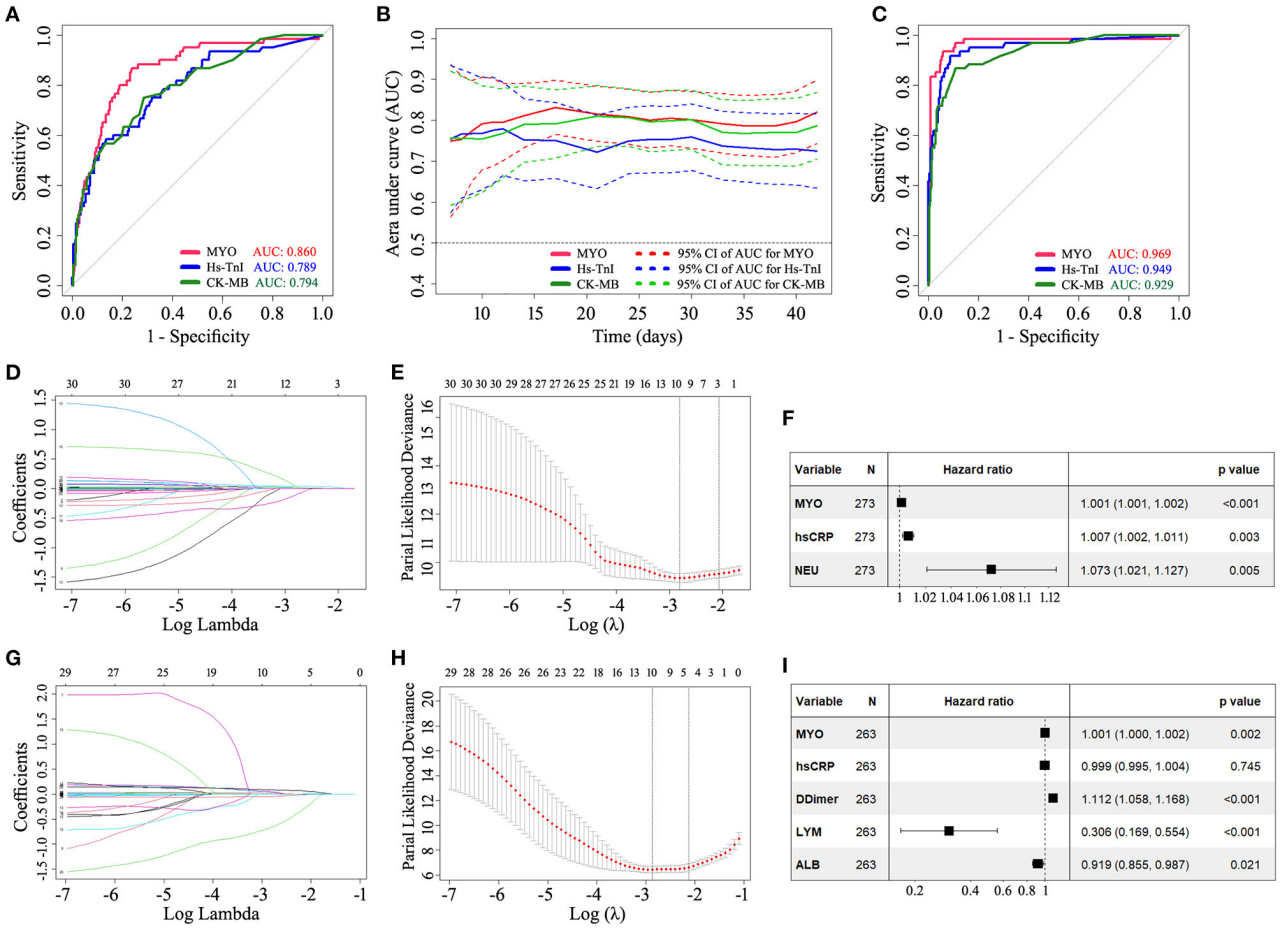
Subgroup analysis demonstrating the prognostic ability of MYO in COVID-19 patients with severe or critical conditions. The results confirmed that MYO provided a better prognostic performance than hs-TnI and CK-MB and had an independent prognostic effect on in-hospital mortality. **(A)** Standard ROC curve analysis based on the early-stage levels of biomarkers. **(B)** Time-dependent AUC curves based on the early levels of cardiac markers. **(C)** Standard ROC curve analysis based on the late-stage levels of biomarkers. **(D–F)** LASSO regression and multivariate COX analysis based on early levels of biomarkers. **(G–I)** LASSO regression and multivariate COX analysis based on late levels of biomarkers. LASSO, least absolute shrinkage and selection operator; MYO, myoglobin; NEU, neutrophil; hs-CRP, high sensitivity C-reactive protein; LYM, lymphocyte; ALB, albumin; N, number; AUC, area under curve.

Given that COVID-19 patients with underlying cardiovascular disease are more likely to exhibit elevated myocardial marker levels than patients without cardiovascular disease (6), we performed a subgroup analysis in patients without a history of cardiovascular disease (see Supplementary Figure 4). The results confirmed that MYO provided better prognostic performance than hs-TnI and CK-MB and that MYO was an independent factor associated with in-hospital mortality in COVID-19. We further performed a subgroup analysis of patients with severe or critical conditions. Similarly, the results showed that MYO provided a better prognostic performance with an independent prognostic effect on in-hospital mortality (Figure 5).

## Discussion

The novel finding of this study is that among myocardial biomarkers, MYO had a higher prognostic performance for in-hospital mortality than hs-TnI and CK-MB, which had been largely ignored in previous studies. MYO had the most enormous AUC value among cardiac biomarkers in the ROC analysis. More importantly, MYO, but not hs-TnI or CK-MB, was an independent factor associated with the risk of in-hospital mortality. These results are consistent with multivariate regression analyses in our and others' previous studies showing that elevated serum myoglobin concentrations were an independent predictor of in-hospital mortality in patients with COVID-19 (10, 12–15).

Comparative studies investigating the ability of myocardial biomarkers to predict COVID-19 mortality have been reported sparsely. In a multicenter retrospective study, Qin et al. evaluated the associations and prognostic power of circulating cardiac injury markers with COVID-19 outcomes. They found that increases in MYO had the highest overall performance in predicting the risk of COVID-19 mortality, followed by NT-proBNP, hs-TnI, and CK-MB (21). Herein, we focused on three commonly used cardiac biomarkers, which were tested as an integrated test package for COVID-19 patients at our institution. Analyses based on these tests could help reduce the statistical bias associated with inconsistent sampling times. In addition, we performed subgroup and dynamic analyses of early and late laboratory data and obtained consistent results confirming that MYO provides a better prognostic performance and has an independent prognostic effect on in-hospital mortality.

These findings are interesting because MYO, a non-cardiac-specific biomarker expressed also in skeletal myocytes, provides even higher prognostic accuracy than cardiac-specific biomarkers such as hs-TnI and CK-MB. The origins are unclear by far. Undeniably, elevated MYO together with hs-TnI and CK-MB is indicative of myocardial cell injury after COVID-19. Rhabdomyolysis, a potential late complication of SARS-CoV-2 infection, is another possible mechanism for the elevation of MYO. Rhabdomyolysis has been reported to be an important factor contributing to poor outcomes in COVID-19 patients (22–25). Patients with rhabdomyolysis usually present with acute renal impairment, as evidenced by elevated creatinine levels (26). Our data showed that the MYO levels in COVID-19 patients were significantly correlated with creatinine levels with a correlation coefficient of 0.46. In contrast, the correlation coefficients for hs-TnI and CK-MB with creatinine were 0.18 and 0.16, respectively. These results suggest that MYO may reflect the severity of disease in COVID-19 patients who develop rhabdomyolysis. However, given that the overall prevalence of rhabdomyolysis in COVID-19 patients is only 2.2% (25), the robust prognostic potency of MYO cannot be attributed entirely to the development of rhabdomyolysis. It is more likely that MYO may be a marker of illness reflecting general physiological disturbance including myocardial injury, acute systemic hypoxia, and rhabdomyolysis during COVID-19 infection, and thus has high accuracy in predicting in-hospital mortality.

Another finding of this study is that although the cardiac-specific biomarkers hs-TnI and CK-MB were correlated with in-hospital mortality, they were not independent prognostic in the multivariate COX analysis. By far, the mechanisms underlying elevated cardiac biomarkers after COVID-19 infection are not fully understood. Myocarditis, stress cardiomyopathy, acute heart failure, and direct viral damage were considered potential etiologies (27). Recent literature data show that troponin release is relatively modest and slightly elevated in overall SARS-CoV-2 infected patients. Only 8-12% of positive cases had hs-TnI concentrations higher than ULN (4). In the present study, we obtained comparable results, with 9.9% of patients with hs-TnI levels higher than ULN in the early stage and 7.2% in the late stage. Considering the generally mild myocardial injury in overall COVID-19 patients, the myocardial injury may not be the major cause of poor prognosis in COVID-19 patients.

Given that many patients with severe COVID-19 infection exhibit concomitant elevations in cardiac biomarkers and inflammatory factors such as hs-CRP and IL-6, it is suggested that the myocardial inflammatory response is the underlying pathophysiology (28). The elevation of cardiac biomarkers and other inflammatory biomarkers raises the possibility that this reflects a cytokine storm, which may clinically present as fulminant myocarditis (4, 27, 29). In addition, increased prothrombotic and procoagulant responses following SARS-CoV-2 infection can lead to increased frequency of pulmonary embolism and worsening hypoxemia, leading to cardiac injury and heart failure (4, 30). The present study showed that hs-TnI levels were highly correlated with inflammation-related factors such as hs-CRP and IL-6 and coagulation indicators such as D-dimer, which, together with MYO, were independent predictors of mortality. Therefore, it is reasonable to assume that for most patients with COVID-19, the myocardial injury may be secondary to inflammatory injury and coagulation abnormalities following viral infection.

Despite the values of these findings, our study has some limitations. First, this study is retrospective in nature. The presence of selection bias associated with patient selection cannot be excluded. Second, the level of laboratory variables may be affected by the fact that the time from diagnosis to hospital admission varies among patients. Finally, this is a single-center study. The current sample size is small for the total patients with COVID-19 worldwide. Further studies involving larger populations and multiple centers are needed to confirm the results.

## Data Availability Statement

The original contributions presented in the study are included in the article/Supplementary Materials, further inquiries can be directed to the corresponding author/s.

## Ethics Statement

The studies involving human participants were reviewed and approved by the ethics committee of Tongji hospital, Huazhong University of Science and Technology. Written informed consent for participation was not required for this study in accordance with the national legislation and the institutional requirements.

## Author Contributions

HL conceived the study and wrote the manuscript. J-SY, R-DC, and HL collected the data. R-DC, H-KY, and L-CZ analyzed the data. J-SY and HL modified the manuscript. All authors contributed to the article and approved the submitted version.

## Conflict of Interest

The authors declare that the research was conducted in the absence of any commercial or financial relationships that could be construed as a potential conflict of interest.

## Publisher's Note

All claims expressed in this article are solely those of the authors and do not necessarily represent those of their affiliated organizations, or those of the publisher, the editors and the reviewers. Any product that may be evaluated in this article, or claim that may be made by its manufacturer, is not guaranteed or endorsed by the publisher.

## Supplementary Material

Supplementary Figure 1 **(A,B)** Percentages of patients with myocardial marker levels below the cut-off, between the cut-off and ULN, and above the ULN in early **(A)** and late **(B)** stages. **(C,D)** Percentages of deaths in patients with myocardial marker levels below the cut-off, between the cut-off and ULN, and above the ULN in early **(C)** and late **(D)** stages. ULN, upper limit of normal.

Supplementary Figure 2 Correlation matrix heat map of 16 clinical parameters in early **(A)** and late **(B)** stages of the disease. Spearman's correlation coefficient was used to calculate the correlation of the variables.

Supplementary Figure 3 Kaplan-Meier curves showing the cumulative survival of patients divided by ULN. **(A-C)** Kaplan-Meier curve analysis based on the early-stage levels of myocardial biomarkers. **(D-F)** Kaplan-Meier curve analysis based on the late-stage levels of myocardial biomarkers. ULN, upper limit of normal.

Supplementary Figure 4 Subgroup analysis demonstrating the prognostic ability of MYO in COVID-19 patients without a history of cardiovascular disease. The results showed that MYO provided a better prognostic performance with an independent prognostic effect on in-hospital mortality. **(A)** Standard ROC curve analysis based on the early-stage levels of biomarkers. **(B)** Time-dependent AUC curves based on the early levels of cardiac markers. **(C)** Standard ROC curve analysis based on the late-stage levels of biomarkers. **(D-F)** LASSO regression and multivariate COX analysis based on early levels of biomarkers. **(G-I)** LASSO regression and multivariate COX analysis based on late levels of biomarkers. LASSO, least absolute shrinkage and selection operator; MYO, myoglobin; NEU, neutrophil; hs-CRP, high sensitivity C-reactive protein; IL-6, interleukin 6; *N*, number; AUC, area under curve.

Supplementary Table 1 Laboratory results at the early and late stages in the overall study population, survivors and non-survivors.

Supplementary Table 2 Effects of variables on in-hospital mortality analyzed by univariate Cox proportional-hazards regression.

Supplementary Table 3 Association of elevated myocardial marker levels above ULN with in-hospital mortality of COVID-19.

Supplementary Table 4 Multivariate Cox analysis of prognostic factors identified by LASSO regression.
