# Supplementary figures and images for: Myoglobin Offers Higher Accuracy Than Other Cardiac-Specific Biomarkers for the Prognosis of COVID-19
Source: Front Cardiovasc Med. 2021 Aug 12;8:686328. doi: 10.3389/fcvm.2021.686328 (PMC8387634; doi:10.3389/fcvm.2021.686328)

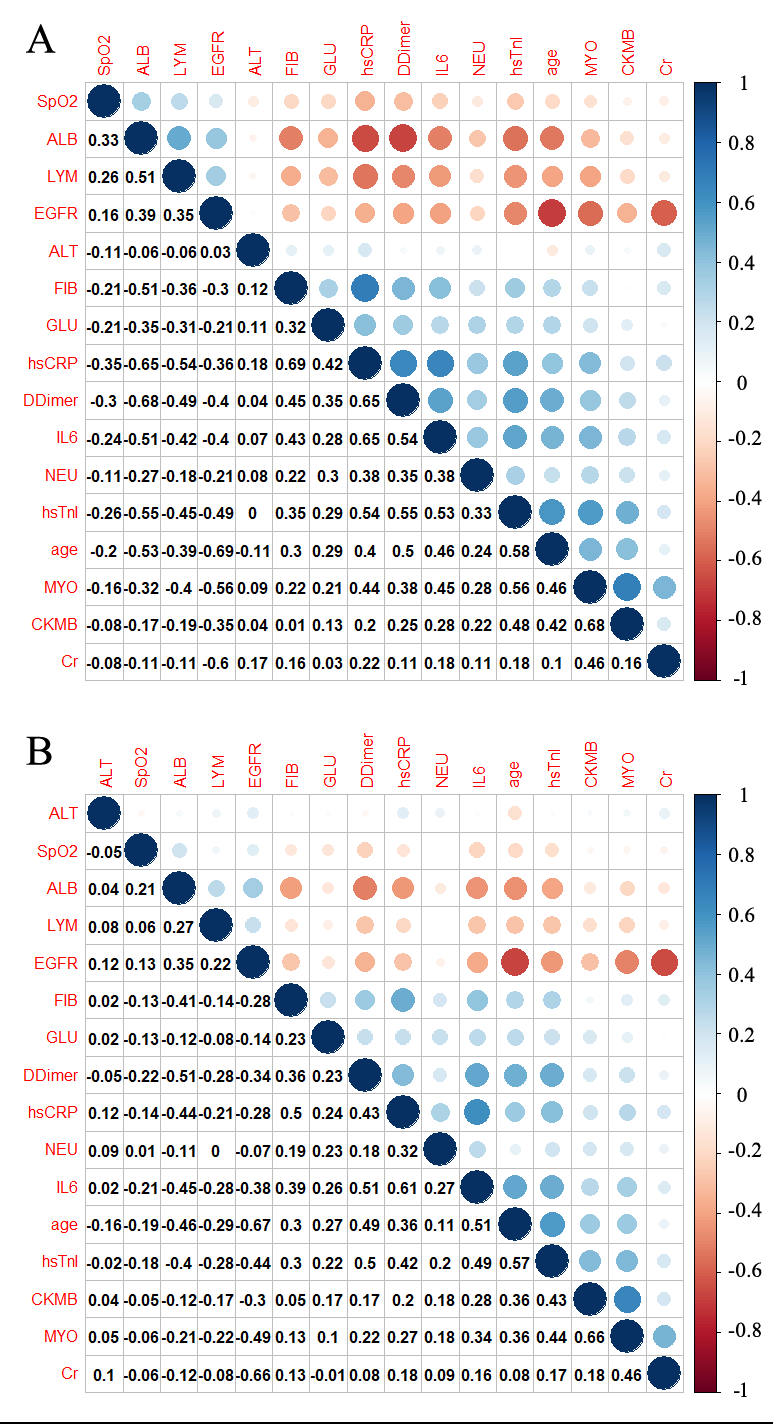

Supplement: Supplementary Figure 2 — Correlation matrix heat map of 16 clinical parameters in early (A) and late (B) stages of the disease. Spearman's correlation coefficient was used to calculate the correlation of the variables. [file Image_2.TIF]

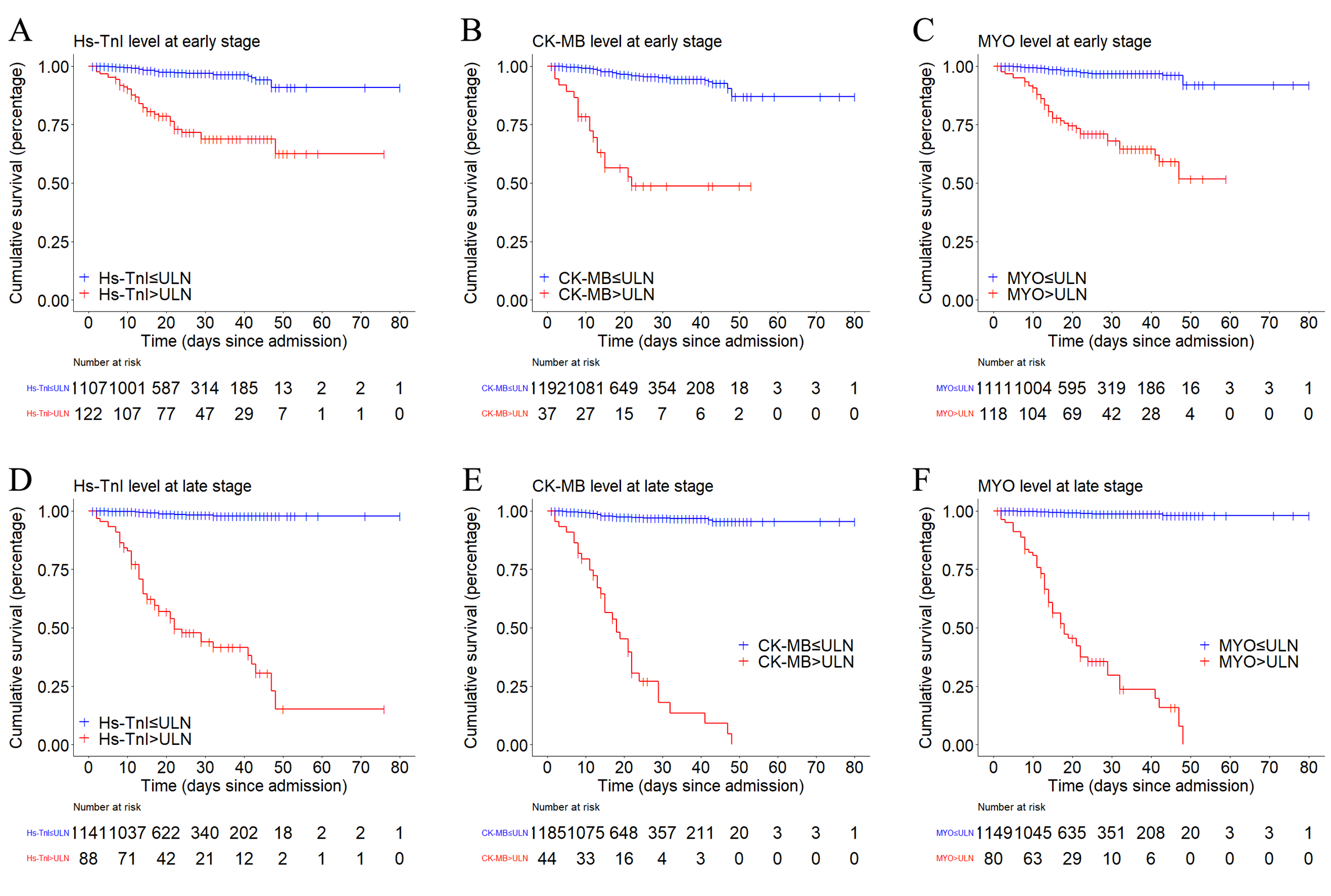

Supplement: Supplementary Figure 3 — Kaplan-Meier curves showing the cumulative survival of patients divided by ULN. (A-C) Kaplan-Meier curve analysis based on the early-stage levels of myocardial biomarkers. (D-F) Kaplan-Meier curve analysis based on the late-stage levels of myocardial biomarkers. ULN, upper limit of normal. [file Image_3.TIF]

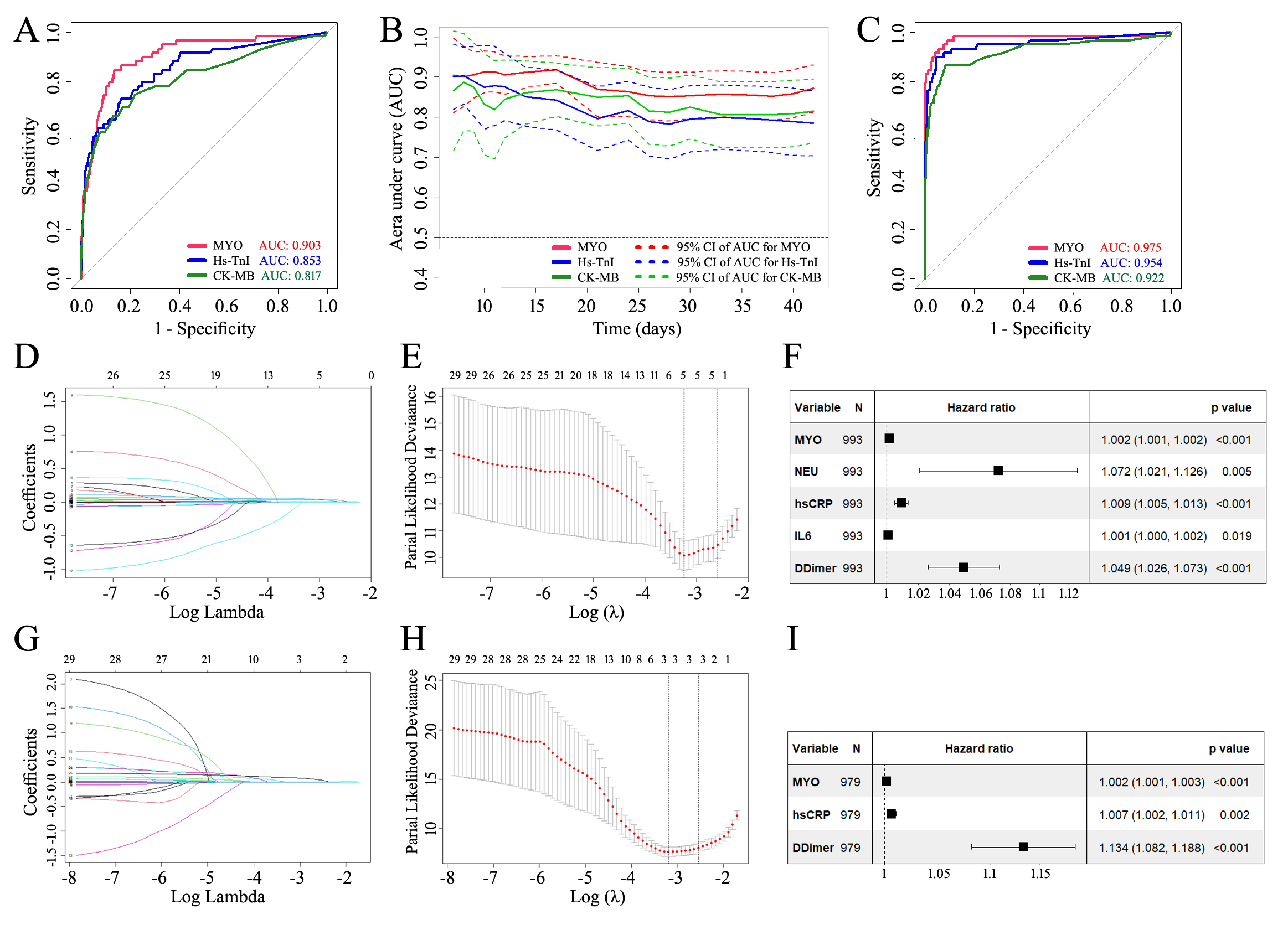

Supplement: Supplementary Figure 4 — Subgroup analysis demonstrating the prognostic ability of MYO in COVID-19 patients without a history of cardiovascular disease. The results showed that MYO provided a better prognostic performance with an independent prognostic effect on in-hospital mortality. (A) Standard ROC curve analysis based on the early-stage levels of biomarkers. (B) Time-dependent AUC curves based on the early levels of cardiac markers. (C) Standard ROC curve analysis based on the late-stage levels of biomarkers. (D-F) LASSO regression and multivariate COX analysis based on early levels of biomarkers. (G-I) LASSO regression and multivariate COX analysis based on late levels of biomarkers. LASSO, least absolute shrinkage and selection operator; MYO, myoglobin; NEU, neutrophil; hs-CRP, high sensitivity C-reactive protein; IL-6, interleukin 6; N, number; AUC, area under curve. [file Image_4.TIF]
