# Supplementary material for: Myoglobin Offers Higher Accuracy Than Other Cardiac-Specific Biomarkers for the Prognosis of COVID-19
Source: Front Cardiovasc Med. 2021 Aug 12;8:686328. doi: 10.3389/fcvm.2021.686328 (PMC8387634; doi:10.3389/fcvm.2021.686328)
Supplement: Supplementary Table 1 — Laboratory results at the early and late stages in the overall study population, survivors and non-survivors. [file Table_1.DOCX]

Supplementary Table 1. Laboratory results at the early and late stages in the overall study population, survivors and non-survivors.

| **Biomarkers** | **All included patients (n = 1229)** | | |  | **Alive (n = 1163)** | | |  | **Died (n = 66)** | | |
| --- | --- | --- | --- | --- | --- | --- | --- | --- | --- | --- | --- |
|  | **Early stage** | **Late stage** | ***p* value** |  | **Early stage** | **Late stage** | ***p* value** |  | **Early stage** | **Late stage** | ***p* value** |
| Hs-TnI (pg/mL) | 3.1 (1.9 - 8.0) | 2.3 (1.9 - 5.8) | **< 0.001** |  | 2.8 (1.9 - 7.4) | 2.2 (1.9 - 5.0) | **< 0.001** |  | 25.8 (8.0 - 223.6) | 144.9 (31.3 - 544.9) | **0.014** |
| CK-MB (ng/mL) | 0.7 (0.5 - 1.2) | 0.6 (0.4 - 1.0) | **< 0.001** |  | 0.7 (0.4 - 1.1) | 0.6 (0.4 - 0.9) | **< 0.001** |  | 2.4 (1.0 - 4.4) | 4.8 (1.9 - 10.2) | **< 0.001** |
| MYO (ng/mL) | 35.6 (26.1 - 59.6) | 30.2 (23.5 - 43.5) | **< 0.001** |  | 34.5 (25.6 - 53.9) | 29.5 (23.1 - 39.9) | **< 0.001** |  | 169.2 (95.7 - 368.5) | 672.7 (279.9 - 1200.0) | **< 0.001** |
| NEU (10^9/L) | 3.68 (2.67 - 5.17) | 3.25 (2.51 - 4.19) | **< 0.001** |  | 3.59 (2.63 - 4.97) | 3.16 (2.49 - 4.03) | **< 0.001** |  | 7.50 (4.91 - 11.87) | 11.09 (6.8 - 17.61) | **0.005** |
| LYM (10^9/L) | 1.34 (0.94 - 1.79) | 1.58 (1.25 - 1.96) | **< 0.001** |  | 1.38 (1.00 - 1.82) | 1.61 (1.29 - 1.99) | **< 0.001** |  | 0.60 (0.43 - 0.88) | 0.44 (0.29 - 0.77) | 0.054 |
| Hs-CRP (mg/L) | 5.2 (1.2 - 37.1) | 1.6 (0.7 - 5.3) | **< 0.001** |  | 4.1 (1.1 - 31.1) | 1.5 (0.6 - 4.2) | **< 0.001** |  | 89.1 (44.9 - 144.5) | 114.7 (72.4 - 198.0) | **0.033** |
| IL6 (pg/mL) | 3.76 (1.76 - 11.92) | 3.14 (1.57 - 7.75) | **< 0.001** |  | 3.50 (1.66 - 9.66) | 2.96 (1.51 - 6.35) | **< 0.001** |  | 58.95 (24.55 - 167.55) | 377.60 (75.19 - 1698.50) | **< 0.001** |
| D-dimer (μg/mL FEU) | 0.56 (0.24 - 1.36) | 0.40 (0.22 - 0.94) | **< 0.001** |  | 0.51 (0.23 - 1.14) | 0.37 (0.22 - 0.79) | **< 0.001** |  | 5.61 (1.71 - 21.00) | 6.68 (3.34 - 16.05) | 0.783 |
| FIB (g/L) | 4.06 (3.20 - 5.41) | 3.59 (2.99 - 4.43) | **< 0.001** |  | 4.02 (3.20 - 5.35) | 3.57 (2.99 - 4.30) | **< 0.001** |  | 4.79 (2.94 - 6.09) | 4.52 (3.02 - 5.84) | 0.361 |
| ALT (U/L) | 21.0 (13.0 - 36.0) | 19.0 (12.0 - 30.0) | **< 0.001** |  | 20.0 (13.0 - 35.0) | 19.0 (12.0 - 30.0) | **< 0.001** |  | 26.0 (17.8 - 44.5) | 29.0 (18.0 - 48.0) | 0.815 |
| ALB (g/L) | 37.4 (33.3 - 41.5) | 39.6 (36.5 - 42.0) | **< 0.001** |  | 37.6 (33.8 - 41.7) | 39.8 (36.8 - 42.1) | **< 0.001** |  | 31.3 (27.8 - 34.6) | 31.1 (28.1 - 34.3) | 0.963 |
| Cr (μmol/L) | 67 (56 - 82) | 68 (57 - 81) | 0.318 |  | 67 (56 - 80) | 67 (57 - 80) | 0.886 |  | 88 (66 - 117) | 121 (76 - 166) | **0.008** |
| EGFR (ml/min/1.73m^2) | 93.2 (79.3 - 103.5) | 93.2 (80.6 - 103.0) | 0.979 |  | 93.7 (80.6 - 104.0) | 93.8 (82.4 - 103.4) | 0.258 |  | 67.4 (49.1 - 89.4) | 47.2 (32.2 - 82.6) | **0.012** |
| GLU (mmol/L) | 5.57 (4.97 - 7.02) | 5.25 (4.79 - 6.29) | **< 0.001** |  | 5.51 (4.94 - 6.83) | 5.21 (4.78 - 6.06) | **< 0.001** |  | 7.24 (5.96 - 10.30) | 8.51 (6.32 - 11.32) | 0.258 |

Data were presented as median (inter-quartile range). *p* values were calculated between the early- and the late-stage groups by Wilcoxon signed-ranks test (two-tailed). Abbreviations: Hs-TnI, high sensitivity troponin-I; CK-MB, creatine kinase-MB; MYO, myoglobin; NEU, neutrophil; LYM, lymphocytes; Hs-CRP, high sensitivity C-reactive protein; IL6, interleukin 6; FIB, fibrinogen; ALT, alanine aminotransferase; ALB, albumin; Cr, creatinine; EGFR, estimated glomerular filtration rate; GLU, glucose.
