# Supplementary material for: Myoglobin Offers Higher Accuracy Than Other Cardiac-Specific Biomarkers for the Prognosis of COVID-19
Source: Front Cardiovasc Med. 2021 Aug 12;8:686328. doi: 10.3389/fcvm.2021.686328 (PMC8387634; doi:10.3389/fcvm.2021.686328)
Supplement: Supplementary Table 2 — Effects of variables on in-hospital mortality analyzed by univariate Cox proportional-hazards regression. [file Table_2.DOCX]

Supplementary Table 2. Effects of variables on in-hospital mortality analyzed by univariate Cox proportional-hazards regression.

|  | **Variables** | | **Crude** | | |  | **Model 1** | | |
| --- | --- | --- | --- | --- | --- | --- | --- | --- | --- |
|  |  |  | **Wald χ^2^** | **HR (95% CI)** | ***p* value** |  | **Wald χ^2^** | **HR (95% CI)** | ***p* value** |
| **Baseline characteristics** | Age, per 10 years | | 31.92 | 1.81 (1.48 - 2.23) | < 0.001 |  | 29.35 | 1.78 (1.45 - 2.20) | < 0.001 |
|  | Male vs. Female | | 15.57 | 3.00 (1.74 - 5.17) | < 0.001 |  | 13.14 | 2.79 (1.60 - 4.86) | < 0.001 |
|  | History of HP | no |  | Ref |  |  |  | Ref |  |
|  |  | yes | 0.46 | 1.19 (0.73 - 1.94) | 0.497 |  | 0.49 | 0.83 (0.48 - 1.41) | 0.485 |
|  | History of DM | no |  | Ref |  |  |  | Ref |  |
|  |  | yes | 0.29 | 1.18 (0.65 - 2.13) | 0.591 |  | 0.00 | 0.98 (0.52 - 1.86) | 0.959 |
|  | History of CLD | no |  | Ref |  |  |  | Ref |  |
|  |  | yes | 0.00 | 1.06 (0.15 - 7.68) | 0.951 |  | 0.02 | 1.15 (0.16 - 8.42) | 0.888 |
|  | History of CHD | no |  | Ref |  |  |  | Ref |  |
|  |  | yes | 0.35 | 1.27 (0.58 - 2.78) | 0.556 |  | 0.01 | 0.96 (0.42 - 2.19) | 0.924 |
|  | Cancer history | no |  | Ref |  |  |  | Ref |  |
|  |  | yes | 0.60 | 1.50 (0.54 - 4.21) | 0.438 |  | 0.88 | 1.63 (0.59 - 4.53) | 0.348 |
|  | Stroke history | no |  | Ref |  |  |  | Ref |  |
|  |  | yes | 3.95 | 2.24 (1.01 - 4.97) | 0.047 |  | 2.21 | 1.85 (0.82 - 4.18) | 0.138 |
|  | History of COPD | no |  | Ref |  |  |  | Ref |  |
|  |  | yes | 0.21 | 1.59 (0.22 - 11.5) | 0.645 |  | 0.02 | 1.14 (0.16 - 8.37) | 0.896 |
|  | History of CKD | no |  | Ref |  |  |  | Ref |  |
|  |  | yes | 6.55 | 3.04 (1.30 - 7.12) | 0.010 |  | 2.53 | 2.08 (0.84 - 5.15) | 0.112 |
| **Physical examination on admission** | Temperature | ≤ 36.9 °C |  | Ref |  |  |  | Ref |  |
|  |  | > 36.9 °C | 6.03 | 1.87 (1.13 - 3.08) | 0.014 |  | 3.63 | 1.65 (0.99 - 2.75) | 0.057 |
|  | Respiratory Rate | ≤ 25/min |  | Ref |  |  |  | Ref |  |
|  |  | > 25/min | 16.11 | 2.85 (1.71 - 4.75) | < 0.001 |  | 17.70 | 3.06 (1.82 - 5.16) | < 0.001 |
|  | Pulse Rate | ≤ 83 /min |  | Ref |  |  |  | Ref |  |
|  |  | > 83 /min | 1.12 | 1.34 (0.78 - 2.29) | 0.289 |  | 1.54 | 1.41 (0.82 - 2.44) | 0.214 |
|  | SBP | > 133 mmHg |  | Ref |  |  |  | Ref |  |
|  |  | ≤ 133 mmHg | 2.21 | 0.68 (0.41 - 1.13) | 0.137 |  | 7.30 | 0.48 (0.28 - 0.82) | 0.007 |
|  | DBP | > 72 mmHg |  | Ref |  |  |  | Ref |  |
|  |  | ≤ 72 mmHg | 0.45 | 0.83 (0.49 - 1.42) | 0.503 |  | 0.17 | 0.89 (0.52 - 1.53) | 0.679 |
|  | SpO_2_ | > 94 % |  | Ref |  |  |  | Ref |  |
|  |  | ≤ 94 % | 47.19 | 5.68 (3.46 - 9.35) | < 0.001 |  | 41.55 | 5.18 (3.15 - 8.55) | < 0.001 |
| **Laboratory results at early stage** | Hs-TnI, per 100 pg/mL | | 25.44 | 1.02 (1.01 - 1.03) | < 0.001 |  | 20.47 | 1.02 (1.01 - 1.02) | < 0.001 |
|  | CK-MB, per 1 ng/mL | | 49.89 | 1.08 (1.06 - 1.11) | < 0.001 |  | 17.10 | 1.06 (1.03 - 1.09) | < 0.001 |
|  | MYO, per 100 ng/mL | | 119.40 | 1.33 (1.27 - 1.40) | < 0.001 |  | 70.51 | 1.28 (1.21 - 1.36) | < 0.001 |
|  | NEU, per 1*10^9/L | | 128.79 | 1.18 (1.15 - 1.22) | < 0.001 |  | 100.39 | 1.21 (1.17 - 1.26) | < 0.001 |
|  | LMY, per 0.1*10^9/L | | 57.14 | 0.78 (0.73 - 0.83) | < 0.001 |  | 33.63 | 0.81 (0.75 - 0.87) | < 0.001 |
|  | Hs-CRP, per 10 mg/L | | 126.79 | 1.17 (1.14 - 1.20) | < 0.001 |  | 77.98 | 1.15 (1.11 - 1.19) | < 0.001 |
|  | IL6, per 10 pg/mL | | 73.65 | 1.02 (1.01 - 1.02) | < 0.001 |  | 46.59 | 1.02 (1.01 - 1.03) | < 0.001 |
|  | D-Dimer, per 1 μg/mL FEU | | 104.21 | 1.07 (1.06 - 1.08) | < 0.001 |  | 71.89 | 1.07 (1.05 - 1.09) | < 0.001 |
|  | FIB, per 1 g/L | | 0.12 | 1.03 (0.88 - 1.19) | 0.733 |  | 1.46 | 0.91 (0.77 - 1.06) | 0.227 |
|  | ALB, per 10 g/L | | 34.18 | 0.30 (0.20 - 0.45) | < 0.001 |  | 9.76 | 0.49 (0.31 - 0.77) | 0.002 |
|  | ALT, per 10 U/L | | 18.01 | 1.02 (1.01 - 1.02) | < 0.001 |  | 7.90 | 1.01 (1.00 - 1.02) | 0.005 |
|  | Cr, per 10 μmol/L | | 33.57 | 1.04 (1.03 - 1.05) | < 0.001 |  | 17.33 | 1.04 (1.02 - 1.05) | < 0.001 |
|  | EGFR, per 10 ml/min/1.73m^2 | | 59.46 | 0.71 (0.66 - 0.78) | < 0.001 |  | 19.99 | 0.77 (0.69 - 0.86) | < 0.001 |
|  | GLU, per 1 mmol/L | | 26.85 | 1.10 (1.06 - 1.15) | < 0.001 |  | 24.02 | 1.16 (1.09 - 1.23) | < 0.001 |
| **Laboratory results at late stage** | Hs-TnI, per 100 pg/mL | | 67.53 | 1.06 (1.05 - 1.08) | < 0.001 |  | 32.00 | 1.04 (1.03 - 1.06) | < 0.001 |
|  | CK-MB, per 1 ng/mL | | 44.49 | 1.02 (1.02 - 1.03) | < 0.001 |  | 34.84 | 1.03 (1.02 - 1.04) | < 0.001 |
|  | MYO, per 100 ng/mL | | 253.96 | 1.41 (1.35 - 1.47) | < 0.001 |  | 171.17 | 1.38 (1.31 - 1.45) | < 0.001 |
|  | NEU, per 1*10^9/L | | 160.89 | 1.12 (1.10 - 1.14) | < 0.001 |  | 88.06 | 1.13 (1.10 - 1.16) | < 0.001 |
|  | LMY, per 0.1*10^9/L | | 2.60 | 0.97 (0.92 - 1.01) | 0.107 |  | 0.04 | 1.00 (0.98 - 1.03) | 0.840 |
|  | Hs-CRP, per 10 mg/L | | 280.87 | 1.18 (1.16 - 1.21) | < 0.001 |  | 178.38 | 1.19 (1.16 - 1.22) | < 0.001 |
|  | IL6, per 10 pg/mL | | 169.30 | 1.01 (1.01 - 1.01) | < 0.001 |  | 114.84 | 1.01 (1.01 - 1.01) | < 0.001 |
|  | D-Dimer, per 1 μg/mL FEU | | 279.84 | 1.26 (1.23 - 1.29) | < 0.001 |  | 229.00 | 1.28 (1.24 - 1.32) | < 0.001 |
|  | FIB, per 1 g/L | | 14.20 | 1.36 (1.16 - 1.60) | < 0.001 |  | 4.04 | 1.20 (1.00 - 1.42) | 0.044 |
|  | ALB, per 10 g/L | | 88.93 | 0.10 (0.07 - 0.17) | < 0.001 |  | 54.86 | 0.14 (0.08 - 0.23) | < 0.001 |
|  | ALT, per 10 U/L | | 22.06 | 1.01 (1.01 - 1.01) | < 0.001 |  | 26.52 | 1.01 (1.01 - 1.02) | < 0.001 |
|  | Cr, per 10 μmol/L | | 132.09 | 1.08 (1.07 - 1.10) | < 0.001 |  | 67.16 | 1.07 (1.05 - 1.08) | < 0.001 |
|  | EGFR, per 10 ml/min/1.73m^2 | | 133.72 | 0.63 (0.58 - 0.68) | < 0.001 |  | 93.11 | 0.62 (0.56 - 0.68) | < 0.001 |
|  | GLU, per 1 mmol/L | | 146.28 | 1.21 (1.17 - 1.25) | < 0.001 |  | 100.45 | 1.25 (1.19 - 1.30) | < 0.001 |

Model 1: Adjusted for age, sex, and co-existing diseases (hypertension, diabetes, coronary heart disease, chronic obstructive pulmonary disease, chronic liver disease, stroke history, chronic kidney disease, and cancer history)

Abbreviations: HR, hazard ratio; CI, confidence interval; HP, hypertension; DM, diabetes; CHD, coronary heart disease; COPD, chronic obstructive pulmonary disease; SBP, systolic blood pressure; DBP, diastolic blood pressure; SpO_2_, percutaneous oxygen saturation; Hs-TnI, high sensitivity troponin-I; CK-MB, creatine kinase-MB; MYO, myoglobin; NEU, neutrophil; LYM, lymphocytes; Hs-CRP, high sensitivity C-reactive protein; IL6, interleukin 6; FIB, fibrinogen; ALT, alanine aminotransferase; ALB, albumin; Cr, creatinine; EGFR, estimated glomerular filtration rate; GLU, glucose.
