# Supplementary material for: Myoglobin Offers Higher Accuracy Than Other Cardiac-Specific Biomarkers for the Prognosis of COVID-19
Source: Front Cardiovasc Med. 2021 Aug 12;8:686328. doi: 10.3389/fcvm.2021.686328 (PMC8387634; doi:10.3389/fcvm.2021.686328)
Supplement: Supplementary Table 3 — Association of elevated myocardial marker levels above ULN with in-hospital mortality of COVID-19. [file Table_3.DOCX]

Supplementary Table 3. Association of elevated myocardial marker levels above ULN with in-hospital mortality of COVID-19.

|  | **Biomarkers** | **Crude** | |  | **Model 1** | |
| --- | --- | --- | --- | --- | --- | --- |
|  |  | **HR (95% CI)** | ***p* value** |  | **HR (95% CI)** | ***p* value** |
| **Early-stage levels** | **Hs-TnI** |  |  |  |  |  |
|  | ≤ ULN | Ref |  |  | Ref |  |
|  | > ULN | 8.64 (5.31 - 14.06) | < 0.001 |  | 7.47 (4.39 - 12.72) | < 0.001 |
|  | **CK-MB** |  |  |  |  |  |
|  | ≤ ULN | Ref |  |  | Ref |  |
|  | > ULN | 13.75 (7.89 - 23.94) | < 0.001 |  | 10.37 (5.87 - 18.30) | < 0.001 |
|  | **MYO** |  |  |  |  |  |
|  | ≤ ULN | Ref |  |  | Ref |  |
|  | > ULN | 12.16 (7.45 - 19.83) | < 0.001 |  | 7.96 (4.75 - 13.35) | < 0.001 |
| **Late-stage levels** | **Hs-TnI** |  |  |  |  |  |
|  | ≤ ULN | Ref |  |  | Ref |  |
|  | > ULN | 45.47 (25.88 - 79.88) | < 0.001 |  | 36.35 (20.13 - 65.66) | < 0.001 |
|  | **CK-MB** |  |  |  |  |  |
|  | ≤ ULN | Ref |  |  | Ref |  |
|  | > ULN | 38.67 (23.75 - 62.98) | < 0.001 |  | 27.37 (16.19 - 46.28) | < 0.001 |
|  | **MYO** |  |  |  |  |  |
|  | ≤ ULN | Ref |  |  | Ref |  |
|  | > ULN | 90.28 (48.15 - 169.3) | < 0.001 |  | 77.54 (39.09 - 153.82) | < 0.001 |

The variables were categorized into two groups according to ULN. The biomarkers were included as dichotomous variables in the univariable COX regression analysis.

Model 1: Adjusted for age, sex, and co-existing diseases (hypertension, diabetes, coronary heart disease, chronic obstructive pulmonary disease, chronic liver disease, stroke history, chronic kidney disease, and cancer history)

Abbreviations: ULN, upper limit of normal; HR, hazard ratio; CI, confidence interval; Hs-TnI, high sensitivity troponin-I; CK-MB, creatine kinase-MB; MYO, myoglobin.
