# Supplementary material for: Myoglobin Offers Higher Accuracy Than Other Cardiac-Specific Biomarkers for the Prognosis of COVID-19
Source: Front Cardiovasc Med. 2021 Aug 12;8:686328. doi: 10.3389/fcvm.2021.686328 (PMC8387634; doi:10.3389/fcvm.2021.686328)
Supplement: Supplementary Table 4 — Multivariate Cox analysis of prognostic factors identified by LASSO regression. [file Table_4.DOCX]

Supplementary Table 4. Multivariate Cox analysis of prognostic factors identified by LASSO regression.

|  | **N** | **Deaths** | **Elected variables** | **β** | **HR (95% CI)** | ***p* value** | ***C*-index (SE)** |
| --- | --- | --- | --- | --- | --- | --- | --- |
| **Multivariate COX model based on early-stage biomarker levels** | 1078 | 61 | MYO | 0.00149 | 1.001 (1.001-1.002) | < 0.001 | 0.922 (0.013) |
|  |  |  | NEU | 0.0879 | 1.092 (1.044-1.143) | < 0.001 |  |
|  |  |  | Hs-CRP | 0.00849 | 1.009 (1.005-1.013) | < 0.001 |  |
|  |  |  | IL-6 | 0.00121 | 1.001 (1.001-1.002) | < 0.001 |  |
|  |  |  | D-Dimer | 0.0411 | 1.042 (1.021-1.064) | < 0.001 |  |
| **Multivariate COX model based on late-stage biomarker levels** | 1063 | 54 | MYO | 0.00180 | 1.002 (1.001-1.003) | < 0.001 | 0.974 (0.004) |
|  |  |  | Hs-CRP | 0.00863 | 1.009 (1.005-1.012) | < 0.001 |  |
|  |  |  | D-Dimer | 0.121 | 1.128 (1.082-1.177) | < 0.001 |  |

The potential prognostic factors included in the multivariate models were screened by the least absolute shrinkage and selection operator (LASSO) regression. Variables were included as continuous variables in the multivariate COX analysis.

Abbreviations: N, number; C-index, concordance index; SE, standard error; HR, hazard ratio; CI, confidence interval; MYO, myoglobin; NEU, neutrophil; Hs-CRP, high sensitivity C-reactive protein; IL-6, interleukin 6.
